# ERG overexpression plus SLC45A3 (prostein) and PTEN expression loss: Strong association of the triple hit phenotype with an aggressive pathway of prostate cancer progression

**DOI:** 10.18632/oncotarget.18266

**Published:** 2017-05-26

**Authors:** Silvia Hernández-Llodrà, Nuria Juanpere, Silvia de Muga, Marta Lorenzo, Joan Gil, Alba Font-Tello, Laia Agell, Raquel Albero-González, Laura Segalés, José Merino, Laia Serrano, Lluís Fumadó, Lluís Cecchini, Josep Lloreta-Trull

**Affiliations:** ^1^ Department of Health and Experimental Sciences, Universitat Pompeu Fabra, Barcelona, Spain; ^2^ Department of Pathology, Hospital del Mar-Parc de Salut Mar-IMIM, Barcelona, Spain; ^3^ Dana Farber Cancer Institute, Boston, MA, USA; ^4^ Fundación Jiménez Díaz, Madrid, Spain; ^5^ Department of Urology, Hospital del Mar-Parc de Salut Mar-IMIM, Barcelona, Spain

**Keywords:** ERG, SLC45A3, PTEN, prostate cancer, progression

## Abstract

*TMPRSS2* and *SLC45A3* rearrangements may coexist in the same tumor. *ERG* rearrangements and *PTEN* loss are concomitant events in prostate cancer (PrCa), and can cooperate in progression. We have reported that mRNA expression of *TMPRSS2-ERG* and *SLC45A3-ERG* rearrangements plus *PTEN* loss define an aggressive tumor subset. The aim of this study has been to validate these results by immunohistochemistry in a large cohort of tumors. ERG, SLC45A3 and PTEN immunostaining and their association with pathological features and PSA progression-free survival were analyzed in 220 PrCa (PSMAR-Biobank, Barcelona, Spain). ERG protein expression was found in 46.8% and SLC45A3 and PTEN loss in 30% and 34% tumors, respectively. Single ERG positive immunostaining was associated with GS = 6 tumors (*p* = 0.016), double ERG+/PTEN loss with GS = 7 (*p* = 0.008) and Grade Group 2 (GG) or GG3 cases (*p* = 0.042), ERG+/SLC45A3 loss/PTEN loss (“triple hit”) with GS ≥ 8 (*p* < 0.0001) and GG4 or GG5 tumors (*p* = 0.0003). None of GS = 6 nor = GG1 cases showed this combination. In the GS ≥ 8 group, ERG+ (*p* = 0.002), PTEN loss (*p* = 0.009) and “triple hit” (*p* = 0.003) were associated with Gleason pattern 3 component, and single SLC45A3 loss (*p* = 0.036) with GS ≥ 8 without pattern 3. The number of aberrant events and the triple hit were strongly associated with shorter PSA progression-free survival. In GS = 6 PrCa, single ERG+ was also associated with progression. ERG+ identifies a distinct pathway of PrCa. Additional assessment of PTEN and SLC45A3 adds relevant prognostic information. The triple hit phenotype (ERG+/SLC45A3 loss/PTEN loss) is associated with progression and could be used for patient stratification, treatment and follow-up.

## INTRODUCTION

ERG overexpression resulting from the rearrangement between the *TMPRSS2* promoter and the coding regions of the *ERG* gene has been identified as the most common alteration in prostate cancer (PrCa), accounting for more than 90% of the *TMPRSS2-ETS* family rearrangements [1–3]. *TMPRSS2* is also fused with lower frequency to other ETS members, and less frequent 5′partners, such as *SLC45A3*, *NDRG1* and others have been found [4–8]. Among them, *SLC45A3* stands up as the second most common 5′ partner gene in *ERG* rearrangements [7, 9–11]. Furthermore, double rearrangements of *ERG* with *TMPRSS2* and *SLC45A3* or *NDRG1,* well known androgen-induced genes (8), have been found to coexist in the same tumor in a subset of PrCa cases [9–11].

*TMPRSS2-ERG* has been reported as an early event in prostate carcinogenesis, but there is still controversy about its role in the development and progression of PrCa [11–20]. On the other hand, PTEN loss is a well characterized and crucial step in the pathogenesis of PrCa. The association of PTEN loss with high grade prostate tumors is well documented [21–24]. Both *ERG* rearrangements and loss of *PTEN* are frequent and concomitant events in PrCa that can cooperate in progression [11, 14, 20, 25–28]. PTEN loss has been considered a subclonal event, consecutive to *ERG* gene fusion [29, 30]. In Gleason score (GS) 6 biopsies, PTEN loss has been proposed as a potential predictor of upgrading at radical prostatectomy [31, 32].

Several papers have addressed the possible role of the *SLC45A3-ERG* rearrangement in PrCa [7–10, 33]. Some authors have not found any association between this rearrangement and the clinical-pathological features of the tumors or their prognosis [9, 10]. On the other hand, Perner *et al.* [33] have reported that loss of the SLC45A3 protein (also known as prostein) as a result of the *SLC45A3-ERG* rearrangement is associated with shorter PSA progression-free survival and high GS.

In that sense, we have recently reported that expression levels of *TMPRSS2-ERG* fusion and *ERG* mRNA, rather than their mere presence, are related to a more aggressive PrCa phenotype [20]. Moreover, previous studies from our group have shown that *TMPRSS2-ERG* is associated with low grade PrCa only when it is the single rearrangement present, and also that *SLC45A3-ERG* is more likely to appear as a second fusion in cases already harboring the *TMPRSS2-ERG* fusion. This second rearrangement, as well as *PTEN* loss could mark the transition to higher grade and stage [11]. Interestingly, we have not found coexisting *TMPRSS2-ERG*, *SLC45A3-ERG* plus *PTEN* loss in low grade or low stage PrCa. This combination of three molecular events (“triple hit”) defines an aggressive tumor subset, and could have potential impact in patient management.

The aim of the present study has been to analyze by immunohistochemistry (IHC) the expression of ERG, SLC45A3 and PTEN in a large, independent cohort of PrCa samples with the purpose of replicating our previous results and assessing their potential relationship with the clinical-pathological variables and with PSA progression-free survival.

## RESULTS

### Immunostaining analysis of ERG, SLC45A3 and PTEN in the TMAs

From the 220 cases included in the TMA, we had more than one core in 182 cases. Ninety-two of these cases had all the same GS classification in all cores, whereas in the other 90 cases, there were cores with different GS classification. In all cases, the core with the highest grade was representative of the global GS of the patient. The concordance between the immunostaining results of ERG, SLC45A3 and PTEN through the different cores from the same patients were 85.8%, 72.5% and 74.7%, respectively. ERG expression showed the highest concordance values, being higher between cores with the same GS (94.6%) than between cores with different GS (76.7%). In the SLC45A3 and PTEN expression analysis, concordance was also higher in cores with the same GS (80.4% and 89.1%) than in cores with different GS (64.4% and 60%). The core with the highest grade and the highest number of alterations was selected to analyse the relationship between ERG, SLC45A3 and PTEN expression and the clinical-pathological parameters of the cases.

### Relationship between ERG, SLC45A3 and PTEN expression in PrCa

**Figure 1:**
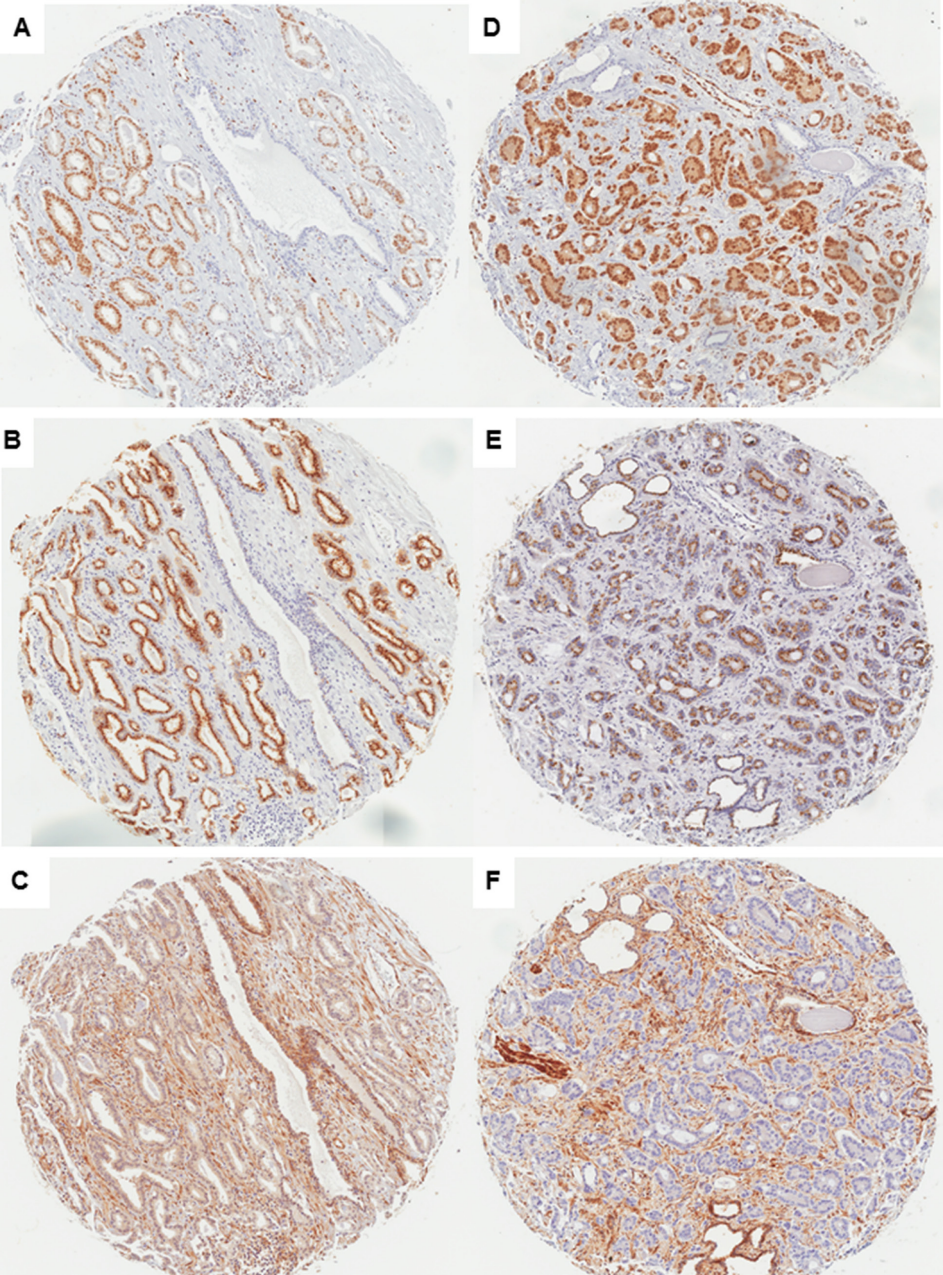
Immunostaining of ERG, SLC45A3 (prostein) and PTEN in PrCa (×50) Case 1, GS = 6 (GG1) tumor: ERG positive immunostaining (**A**), SLC45A3 fully positive immunostaining (**B**), and PTEN fully positive immunostaining (**C**). Case 2, GS ≥ 8 (GG5) tumor: ERG positive immunostaining (**D**), SLC45A3 partial expression loss (**E**) and marked PTEN expression loss (**F**).

ERG positive immunostaining was found in 103 tumor samples (46.8%), and it was absent in 117 (53.2%). Loss of SLC45A3 expression, considered for scores 0 (*n* = 3) and 1 (*n* = 63), was detected in 66 cases (30%). PTEN loss was considered for scores 0 (*n* = 25) and 1 (*n* = 50), and was detected in 75 cases (34%) (Figure 1).

Among cases with positive ERG immunostaining, 56 (54.4%) showed full PTEN expression, while total or partial PTEN expression loss was found in 47 (45.6%). On the other hand, among cases without ERG expression, 89 (76%) expressed PTEN and 28 (24%) did not. Thus, loss of PTEN expression was statistically associated with *TMPRSS2-ERG* rearrangement-related ERG overexpression (Pearson Chi-Square, *p* = 0.0007).

In addition, among cases with positive ERG immunostaining, 64 (62.1%) had preserved SLC45A3 expression and 39 (37.9%) presented with total or partial loss of SLC45A3 expression. On the other hand, among cases without ERG expression, 90 (76.9%) expressed SLC45A3 and 27 (23%) did not (Pearson Chi-Square, *p* = 0.017). Thus, decreased SLC45A3 expression, probably related to a *SLC45A3*-*ERG* rearrangement, is statistically more frequent in ERG positive tumors.

Finally, there was also a statistical association between SLC45A3 and PTEN expression; 111 of 154 (72%) PTEN positive cases were also positive for SLC45A3, and 32 of 66 (48.5%) cases with PTEN loss had also loss of SLC45A3 protein expression (Pearson Chi-Square, *p* = 0.003).

### Relationship between FISH and immunohistochemical analysis of SLC45A3

To correlate the immunostaining pattern of SLC45A3 with the genetic status of *SLC45A3* gene, FISH and immunohistochemistry analyses were carried out in an independent series of 37 PrCa whole sections. *SLC45A3* rearrangement was detected in 20/37 cases studied by FISH. On the other hand, 25/37 tumors were considered to have loss of protein expression, with areas of 1+ or 0 score. The 12 remaining cases showed an homogeneous normal pattern of SLC45A3 expression (2+). Comparing FISH and immunostaining, there were only 6 cases with discordant results between both techniques (κ = 0.667). Sensitivity, specificity, PPV and NPV were: 79.2%, 91.7%, 95% and 68.7% respectively.

### Single and combined ERG, SLC45A3 and PTEN alterations and relationship with the clinical-pathological features of PrCa

Distribution of total alterations according to the combined GS and the Grade Group (GG) classifications is shown in Table 1. Decreased expression of SLC45A3 and also of PTEN was statistically associated with higher GS and with higher PrCa GG.

**Table 1:** Total ERG, SLC45A3 and PTEN expression alterations according GS and GG

ERG, SLC45A3 and PTEN expression alterations according to GS and GG (WHO-ISUP 2016)						
Type of alteration	GS = 6 Tumors	GS = 7 Tumors		GS ≥ 8 Tumors		*P*-value
**Total ERG+**	15 (38.5%)	50 (48.5%)		38 (48.7%)		0.514
**Total SLC45A3-**	3 (7.7%)	27 (26.2%)		36 (46.2%)		**0.0005**
**Total PTEN-**	4 (10.3%)	39 (37.9%)		32 (41%)		**0.002**
**Type of alteration**	**GG1 Tumors**	**GG2 Tumors**	**GG3 Tumors**	**GG4 Tumors**	**GG5 Tumors**	***P*-value**
**Total ERG+**	15 (38.5%)	37 (45.7%)	13 (59%)	14 (42.4%)	24 (53.3%)	0.479
**Total SLC45A3-**	3 (7.7%)	21 (25.9%)	7 (31.8%)	12 (36.4%)	24 (53.3%)	**0.0001**
**Total PTEN-**	4 (10.3%)	29 (35.8%)	10 (45.4%)	12 (36.4%)	20 (44.4%)	**0.009**

Considering all three genes (Table 2), 72 tumors had a normal pattern of expression, a single event was detected in 74 cases, two events in 52, and three events, or “triple hit”, in 22 prostate tumors. According to the GS classification, the percentage of cases without immunohistochemical expression alterations in the GS = 6 group was 53.8% (21 of 39) and decreased in higher GS cases (Pearson Chi-square, *p* = 0.0067). The percentage of tumors with one alteration was similar across the different GS subgroups (Pearson Chi-square, *p* = 0.886). According to the type of single alteration (Table 2), single ERG positive immunostaining was found in 30.8% of GS = 6, 14.6% of GS = 7 and in 15.4% of GS ≥ 8 prostate tumor samples (Pearson Chi-Square, *p* = 0.062). As the percentages were very similar in GS = 7 and GS ≥ 8, we also analysed the data combining these two groups in a single one. Thus, considering only two categories, GS = 6 and GS ≥ 7, ERG expression was associated with low GS tumors (Pearson Chi-Square, *p* = 0.018). Neither loss of expression of single SLC45A3 (Pearson Chi-Square, *p* = 0.205) nor of PTEN (Pearson Chi-Square, *p* = 0.284) were associated with any GS subgroup.

**Table 2:** Single and combined alterations according to the different GS categories

ERG, SLC45A3 and PTEN expression alterations and GS					
Type of alteration	Total tumors	GS = 6 Tumors	GS = 7 Tumors	GS ≥ 8 Tumors	*P*-value
**Normal phenotype**	72 (32,7%)	21 (53,8%)	31 (30%)	20 (25,6%)	0.006
**Single ERG+**	39 (17,7%)	12 (30,8%)	15 (14,6%)	12 (15,4%)	0.062
**Single SLC45A3−**	17 (7,7%)	1 (2,6%)	7 (6,8%)	9 (11,6%)	0.205
**Single PTEN−**	18 (8,2%)	1 (2,6%)	11 (10,7%)	6 (7,7%)	0.284
**ERG+/SLC45A3−**	17 (7,7%)	1 (2,6%)	11 (10,7%)	5 (6,4%)	0.233
**SLC45A3−/PTEN−**	10 (4,5%)	1 (2,6%)	4 (3,9%)	5 (6,4%)	0.685
**ERG+/PTEN−**	25 (11,4%)	2 (5,1%)	19 (18,4%)	4 (5,1%)	**0.008**
**Triple hit**	22 (10%)	0	5 (4,8%)	17 (21,8%)	**0.00006**

**Figure 2:**
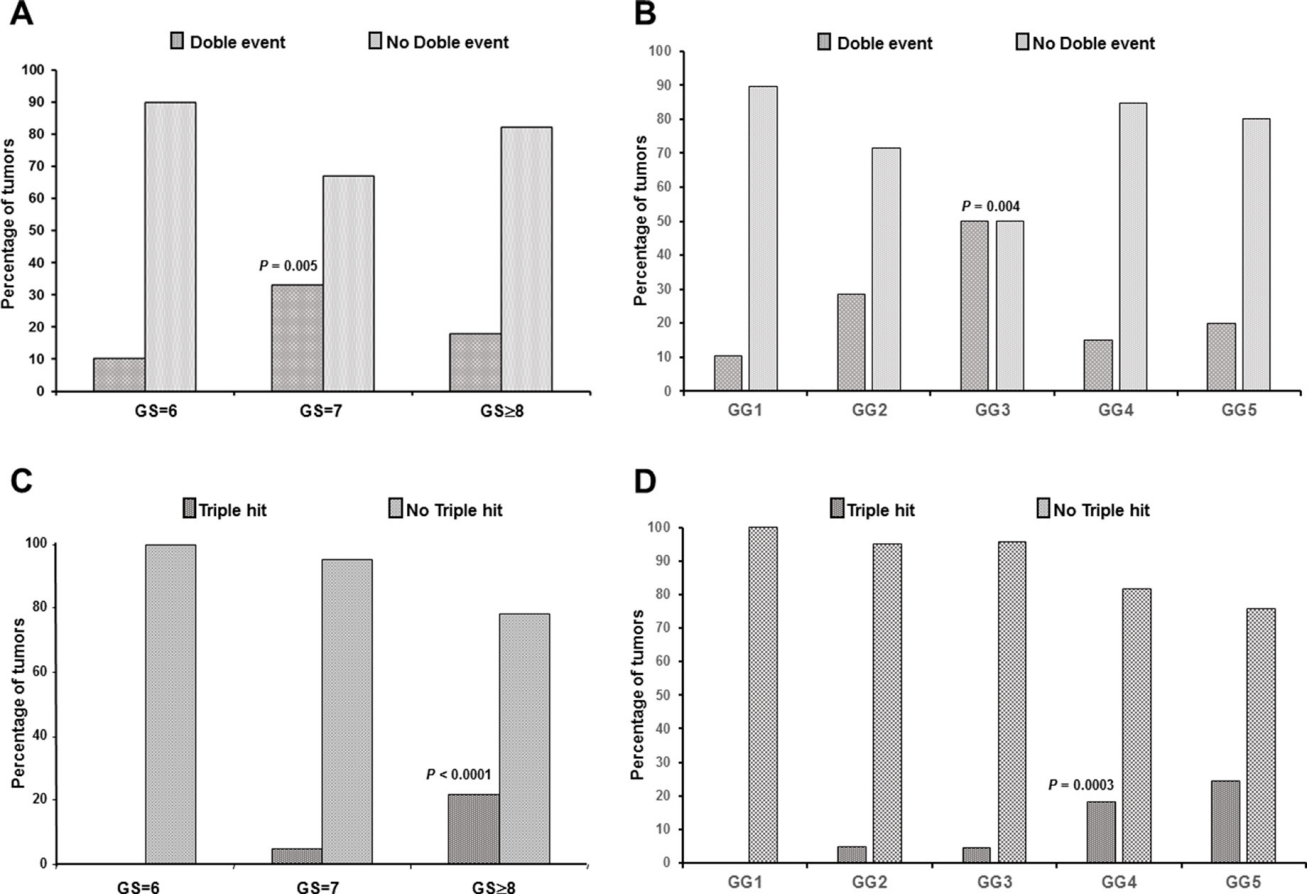
Relationship of double event and “triple hit” (ERG+/SLC45A3 loss/PTEN loss) alterations with GS and GG (**A**) Double event was associated with GS = 7 prostate tumors and (**B**) with GG3 prostate tumors. (**C**) The “triple hit” combination was associated with GS ≥ 8 PrCa and (**D**) with GG4 and GG5 PrCa.

The overall percentage of two alterations was significantly higher in GS = 7 tumors (Pearson Chi-square, *p* = 0.005), but it decreased in the GS ≥ 8 subgroup as a result of the increase in the proportion of the “triple hit” changes in the latter (Figure 2). The combination ERG expression and PTEN loss was statistically associated with GS = 7 prostate tumors (Pearson Chi-Square, *p* = 0.008) (Table 2), as it was detected in 2/39 (5.1%) tumor foci with GS = 6 and 19/103 (18.4%) with GS = 7, but in only 4/78 (5.1%) with GS ≥ 8. On the other hand, concurrent ERG positive/SLC45A3 loss and SLC454A3 loss/PTEN loss phenotypes were not associated with any GS category (Table 2). ERG expression/SLC45A3 loss was detected in only 17 cases, and concurrent SLC454A3 loss/PTEN loss was an infrequent combination.

Finally, the “triple hit” IHC phenotype (ERG expression/ SLC45A3 loss/ PTEN loss) was not detected in any of the GS = 6 prostate tumors, and was found in 5/103 (4.8%) GS = 7, and in 17/78 (21.8%) GS ≥ 8 prostate tumors (Figure 2). Thus, the “triple hit” was statistically associated with high GS prostate tumors (Pearson Chi-Square, *p* < 0.0001).

In addition to the classical standard groups of the GS classification, we have grouped the results using the new WHO-ISUP 2016 proposal (Table 3). Results were very similar to those obtained in the GS tumor classification. Normal phenotype was associated with GG1 tumors (Pearson Chi-square, *p* = 0.007). The overall percentage of tumors with one alteration was similar across the different GGs (Pearson Chi-square, *p* = 0.752), and the percentage of single ERG positive tumors was higher in GG1 but without statistical association (Pearson Chi-Square, *p* = 0.190). Neither loss of expression of single SLC45A3 (Fisher’s exact test, *p* = 0.246) nor of PTEN (Fisher’s exact test, *p* = 0.534) was associated with any of the GGs (Table 3). The overall percentage of two alterations was significantly higher in GG3 tumors (Pearson Chi-square, *p* = 0.046) (Figure 2), and decreased in the GG4 and GG5 subgroups as a result of the increase in the proportion of the “triple hit”. Moreover, the combination of ERG expression and PTEN loss was higher in GG2 and GG3 groups (Pearson Chi-Square, *p* = 0.041), and the triple hit phenotype was higher in GG4 and GG5 prostate tumors (Pearson Chi-Square, *p* = 0.0003) (Figure 2).

**Table 3:** Single and combined alterations according to the different GG categories

ERG, SLC45A3 and PTEN expression alterations and GG (WHO-ISUP 2016)						
Type of alteration	GG1 Tumors	GG2 Tumors	GG3 Tumors	GG4 Tumors	GG5 Tumors	*P*-value
**Normal phenotype**	21 (53,8%)	21 (30,9%)	5 (22,7%)	12 (36,4%)	8 (17,8%)	**0.007**
**Single ERG+**	12 (30,8%)	13 (16%)	2 (9%)	5 (15,1%)	7 (15,5%)	0.190
**Single SLC45A3-**	1 (2,6%)	8 (9,9%)	0	3 (9%)	6 (13,3%)	0.246
**Single PTEN-**	1 (2,6%)	8 (9,9%)	3 (13,6%)	2 (6%)	4 (8,9%)	0.534
**ERG+/SLC45A3−**	1 (2,6%)	6 (7,4%)	5 (2,3%)	1 (3%)	4 (9,76%)	0.080
**SLC45A3-/PTEN-**	1 (2,6%)	3 (3,7%)	1 (4,5%)	2 (6%)	3 (6,7%)	0.864
**ERG+/PTEN−**	2 (5,1%)	14 (17,3%)	5 (22,7%)	2 (6%)	2 (4,4%)	**0.041**
**Triple hit**	0	4 (4,9%)	1 (4,5%)	6 (18,2%)	11 (24,4%)	**0.0003**

### Distribution of the SLC45A3 and PTEN alterations in the ERG positive and in the ERG negative prostate tumors

In order to analyze the potential relationship of SLC45A3 and PTEN alterations with the ERG pathway activation, prostate tumors were divided in 2 groups according to the results of ERG IHC: ERG positive (*n* = 103) and ERG negative (*n* = 117). With regard to the ERG positive cases (Table 4), single ERG expression was strongly associated with GS = 6 tumors (Pearson Chi-Square, *p* = 0.0013). From the GS = 6, ERG positive tumors, 80% (12 of 15) did not show any other alteration. Furthermore, 17 and 25 of the ERG positive prostate tumors, respectively, harbored SLC45A3 or PTEN expression loss. The combination ERG positive/PTEN loss was statistically associated with GS = 7 tumors (Pearson Chi-Square, *p* = 0.006). Finally, in this ERG+ group, the “triple hit” combination was found in 22 prostate tumors: none of the GS = 6 tumors, 5 of 50 (10%) GS = 7 and 17 of 38 GS ≥ 8 (44.8%) prostate tumors showed alterations in the protein expression of all three genes. The “triple hit” IHC phenotype was strongly associated with high GS tumors (Pearson Chi-Square, *p* = 0.00003).

**Table 4:** Single and combined alterations in the ERG+ and ERG− groups according to the different GS categories

Single and combined alterations in the ERG+ group according to the GS				
Type of alteration in the ERG+ tumors (*N* = 103)	GS = 6 Tumors	GS = 7 Tumors	GS ≥ 8 Tumors	*P*-value
**Single ERG+**	12 (80%)	15 (30%)	12 (31,6%)	**0.0013**
**ERG+/SLC45A3−**	1 (6,7%)	11 (22%)	5 (13,1%)	0.292
**ERG+/PTEN−**	2 (13,3%)	19 (38%)	4 (10,5%)	**0.006**
**Triple hit**	0	5 (10%)	17 (44,7%)	**0.00003**
**Single and combined alterations in the ERG- group according to the GS**				
**Type of alteration in the ERG- tumors (*N* = 117)**	**GS = 6 Tumors**	**GS = 7 Tumors**	**GS ≥ 8 Tumors**	***P*-value**
**Normal phenotype**	21 (87,5%)	31(58,5%)	20 (50%)	**0.009**
**Single SLC45A3-**	2 (8,3%)	7 (13,2%)	9 (22,5%)	0.263
**Single PTEN-**	1 (4,2%)	11 (20,7%)	6 (15%)	0.173
**SLC45A3-/PTEN-**	1 (4,2%)	4 (7,5%)	5 (12,5%)	0,576

Regarding the ERG negative cases (Table 4), single PTEN loss was found in 18, single SLC45A3 loss in 18, and the double PTEN/SLC45A3 loss in 10 prostate tumors. These changes were not statistically associated with any GS group. On the other hand, in the ERG negative group, 72 tumors showed SLC45A3 and PTEN protein expression. This phenotype was found in 21/24 (87.5%) of GS = 6, 31/53 (58.5%) of GS = 7 and in 20/40 (50%) of the GS ≥ 8 prostate tumors (Pearson Chi-Square, *p* = 0.009).

### Differences in ERG, SLC45A3 and PTEN expression between GS ≥ 8 cores with or without pattern 3 tumor areas

**Figure 3:**
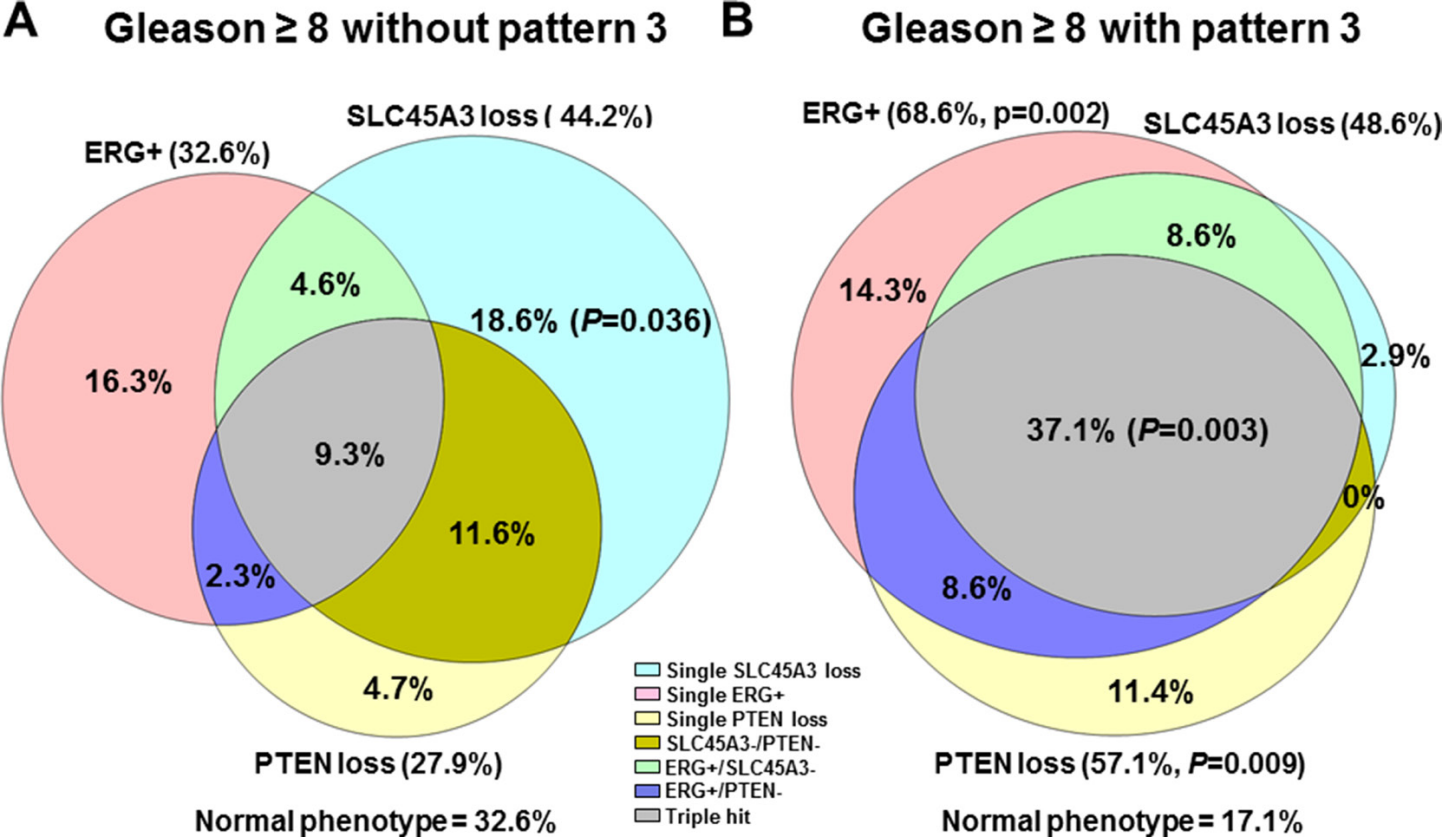
Single and combined ERG, SLC45A3 and PTEN alterations, represented in a Venn’s Diagram in (**A**) GS ≥ 8 PrCa without GS pattern 3 component, and (**B**) GS ≥ 8 PrCa with a pattern 3 area.

Two GS combinations have been compared in the high grade tumor group (GS ≥ 8): cases with a Gleason pattern 3 component (GS ≥ 8 (+3), *n* = 35) and cases without it (*n* = 43) (Figure 3A and 3B).

Total ERG positive immunostaining was statistically more frequent in GS ≥ 8 (+3) cores (24 of 35, 68.6%) than in the GS ≥ 8 cores without pattern 3 area (14 of 43, 32.6%) (Pearson chi-square *p* = 0.002). Furthermore, overall PTEN loss was statistically more frequent in GS ≥ 8 (+3) cores (20 of 35; 57.1%) than in the GS ≥ 8 without pattern 3 cores (12 of 43; 27.9%) (Pearson chi-square *p* = 0.009). Regarding total SLC45A3 expression loss, there were no differences between these two GS subgroups (Pearson chi-square *p* = 0.699). However, single SLC45A3 loss was statistically associated with GS ≥ 8 without pattern 3 (Fisher’s Exact test, *p* = 0.036). The “triple hit” combination was associated with GS ≥ 8 containing pattern 3 areas (Pearson chi-square *p* = 0.003). Other alterations such as single ERG positivity, single PTEN loss, SLC45A3 loss/PTEN loss, ERG positive/PTEN loss, and the normal phenotype were not associated with any of the two GS ≥ 8 categories.

### ERG, SLC45A3 and PTEN expression and PSA progression-free survival analysis in PrCa

**Figure 4:**
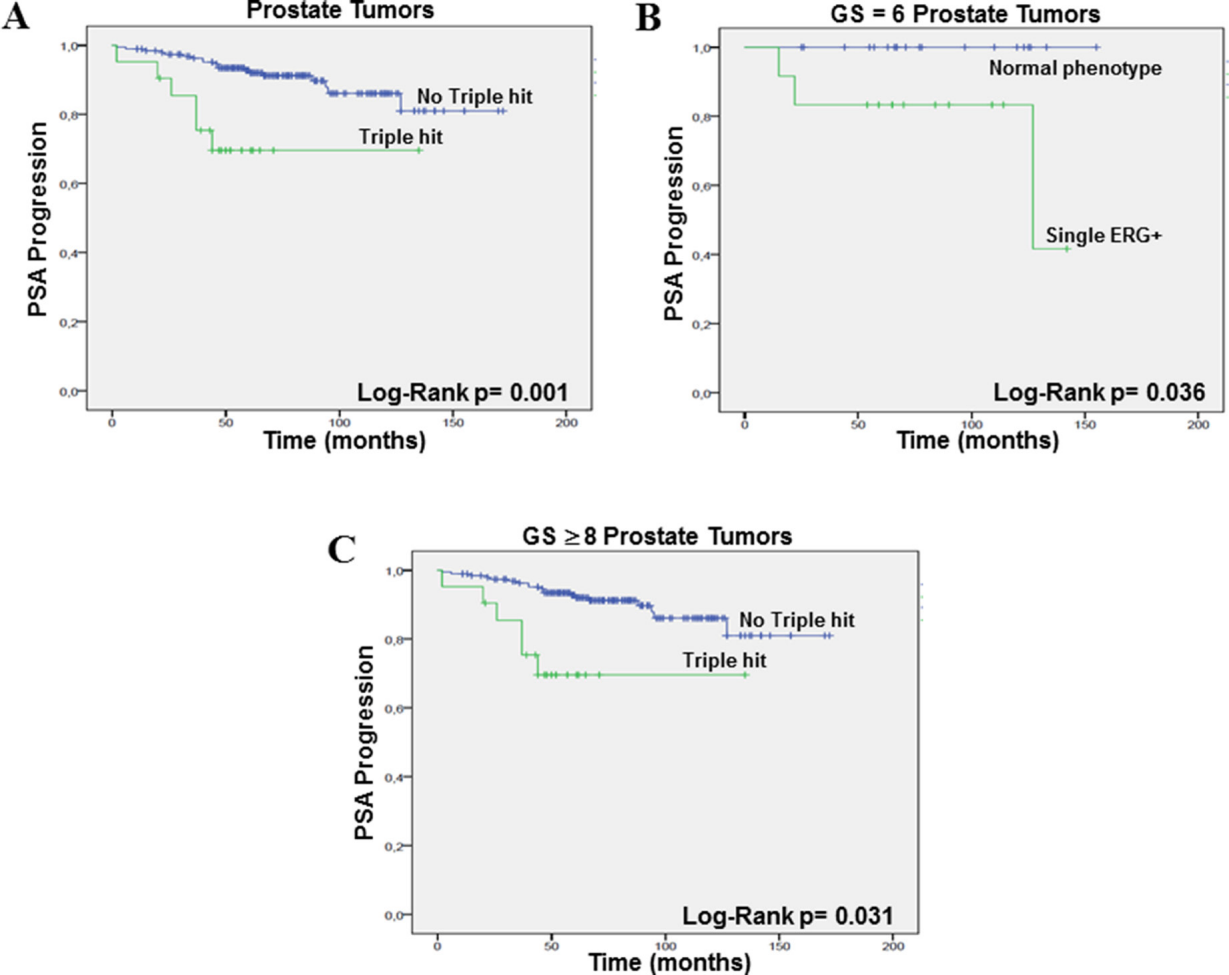
PSA progression-free survival (Kaplan–Meier) plots (**A**) “Triple hit” *vs* no “triple hit”, taking into account all PrCa, (**B**) single ERG+ *vs* triple normal phenotype in the GS = 6 prostate tumors, and (**C**) “triple hit” *vs* no “triple hit” in the GS ≥ 8 PrCa.

A Kaplan-Meier analysis for PSA progression-free survival was performed for GS classification (Log Rank test, *p* = 0.111), ERG positive *vs* ERG negative (Log Rank test, *p* = 0.063), SLC45A3 positive *vs* SLC45A3 loss (Log Rank test, *p* = 0.541), PTEN positive *vs* PTEN loss (Log Rank test, *p* = 0.057), single ERG positive *vs* not single ERG positive (Log Rank test, *p* = 0.800), single SLC45A3 loss *vs* not single SLC45A3 loss (Log Rank test, *p* = 0.190), single PTEN loss *vs* not single PTEN loss (Log Rank test, *p* = 0.428), ERG positive/PTEN loss *vs* not ERG positive/PTEN loss (Log Rank test, *p* = 0.447), number of aberrant events (0, 1, 2 and 3) (Log Rank test, *p* = 0.008), and finally “triple hit” (ERG positive/SLC45A3 loss/PTEN loss) *vs* not “triple hit” (Log Rank test, *p* = 0.001) (Figure 4A). The number of aberrant events and the “triple hit” phenotype were strongly associated with a shorter time of PSA progression free-survival. ERG positive immunostaining (due mostly to an *ERG* rearrangement) and PTEN loss showed a trend to be statistically associated with a shorter time of PSA progression.

In the GS = 6 group, Kaplan-Meier analysis for PSA progression-free survival was performed for ERG positive *vs* ERG negative (Log Rank test, *p* = 0.063), single ERG positive (with normal SLC45A3 and PTEN) *vs* the triple normal phenotype (Log Rank test, *p* = 0.036) (Figure 4B), and ERG positive/PTEN loss *vs* not ERG positive/PTEN loss (Log Rank test, *p* = 0.737). Single ERG alteration was associated with progression in this group of low grade tumors. In the GS = 7 PrCa, analysis was performed for ERG positive *vs* ERG negative (Log Rank test, *p* = 0.212), ERG positive/PTEN loss *vs* not ERG positive/PTEN loss (Log Rank test, *p* = 0.219), and “triple hit” *vs* not “triple hit” (Log Rank test, *p* = 0.102). Finally, in the GS ≥ 8 PrCa group, we analized GS ≥ 8 tumors with and without Gleason pattern 3 areas (Log Rank test, *p* = 0.503), ERG positive *vs* ERG negative (Log Rank test, *p* = 0.578), ERG positive/PTEN loss *vs* not ERG positive/PTEN loss (Log Rank test, *p* = 0.748), and “triple hit” (ERG positive/SLC45A3 loss/PTEN loss) *vs* “not triple hit” (Log Rank test, *p* = 0.031). The “triple hit” phenotype was statistically associated with PSA progression in the high GS PrCa subgroup (Figure 4C).

## DISCUSSION

Rearrangements between promoters of androgen regulated genes and ETS family genes have been identified as the most common alteration in PrCa. The fusion involving *TMPRSS2* and *ERG* accounts for 90% of the cases of fusion-associated PrCa, and the *SLC45A3-ERG* has been reported as the second most frequent type of *ERG* rearrangement in PrCa [3, 5–7, 9–11, 35, 36]. There is still controversy in the literature about the role of *TMPRSS2-ERG* in the development and progression of PrCa [11, 13, 15–20]. Recently, we have reported that it is not the presence of the *TMPRSS2-ERG* fusion but its expression levels and those of *ERG* mRNA what is associated to more aggressive tumors [20].

On the other hand, it has been reported that both *ERG* rearrangements and loss of *PTEN* are frequent and concomitant events that can cooperate in PrCa progression [14, 20, 26–28]. In this regard, a very recent paper [37], has found that *TMPRSS2-ERG* rearrangement and PTEN loss are frequently found in association with heterogeneous loss of DNA repair factors and this has been suggested to reveal an unconventional DNA damage checkpoint regulation mechanism in prostate carcinogenesis, that could have applications in new targeted therapy strategies in this tumor. In addition, the coexistence of *TMPRSS2-ERG* and *SLC45A3-ERG* rearrangements has been identified in a subset of prostate tumors [9–11]. In this regard, we have reported in a recent paper that single *TMPRSS2-ERG* is associated with GS ≤ 7 tumors and double rearrangements with GS ≥ 8 tumors, and also that *SLC45A3-ERG* is more likely to appear as a second fusion in cases already harboring the *TMPRSS2-ERG* rearrangement [11]. In this previous study, the presence of *TMPRSS2-ERG* and *SLC45A3-ERG* rearrangements together with a loss of *PTEN* expression, referred to as “triple hit”, was shown to be strongly associated with higher GS and with T3–4 stage. Thus, assessment of these three molecular changes could impact prognosis and therapeutic decision making in PrCa. In order to validate our previous results, we have analyzed the immunohistochemical expression of ERG, SLC45A3 and PTEN in a large, independent cohort of prostate tumors collected in nine TMA blocks. The concordance between the immunostaining results of ERG, SLC45A3 and PTEN through the different cores from the same patients was very high. Concordance was higher in cores with the same GS than in cores with different GS.

Previous papers have reported a high correlation between *ERG* rearrangements (mainly *TMPRSS2-ERG*) and positive ERG immunohistochemical expression. For *SLC45A3*, a strong inverse association between rearrangement status and immunohistochemical expression of the protein has been shown [33, 38–40]. With regard to the detection of PTEN loss, there is also a good correlation between FISH and immunohistochemistry [21, 31, 41].

In the present study, overall ERG protein expression was found in about 47% of tumors and, in keeping with previous studies, having any kind of alteration involving ERG was not associated with GS [5, 11, 20, 42, 43]. Loss of SLC45A3 and PTEN protein expression were detected in 30% and in 34% of prostate tumors, respectively, and similar to the previous literature, were statistically associated with higher GS [11, 21, 24, 33]. We have analysed the relationship between protein expression of the three genes. Also in concordance with previous studies, loss of PTEN expression was statistically associated with ERG-positive PrCa [11, 14, 20, 25–28]. On the other hand, decreased SLC45A3 expression, usually related to rearrangement, was statistically associated with ERG expression (indicative of *ERG* rearrangement). A statistical relationship between SLC45A3 and PTEN expression was found in our series of PrCa as well.

The distribution of the alterations in the expression of the three genes, either single or combined, was analyzed in the total of 220 PrCa. The normal phenotype was statistically associated with lower GS or GG1 tumors. On the other hand, the percentage of cases with one alteration was similar across the different GS or GG subgroups. A higher percentage of double alterations was statistically associated with GS = 7 and GG3, but this percentage decreased in the GS ≥ 8 and GG4 and GG5 subgroups, most likely as a consequence of the proportional increase in tumors with alterations in the three genes. Interestingly, in keeping with our previous study, the “triple hit” immunohistochemical phenotype (ERG positive/SLC45A3 loss/PTEN loss) was strongly associated with high grade prostate tumors, while this phenotype was completely absent among cases with GS = 6 or GG1.

These findings support our hypothesis that the “triple hit” phenotype could be used as an indicator that cases with GS = 6/GG1 foci in needle biopsy harboring this triple change would be undersampled cases, and that these cases would most likely be upgraded to at least GS = 7 or GG2/GG3 at radical prostatectomy. This fact could have an impact on patient management, as it could be used to select more precisely the optimal candidates for active surveillance.

The most common double alteration was ERG expression/PTEN loss. It was associated with GS = 7 and GG2/GG3 PrCa. Concurrent ERG positive/SLC45A3 loss was detected in only 17 tumors, and loss of SLC45A3 and PTEN expression was, in agreement with our previous results, an infrequent combination never found in low grade tumors [11].

In order to analyze the impact of SLC45A3 and PTEN alterations on ERG pathway activation, prostate tumors were divided in 2 groups depending on ERG immunohistochemical expression being positive (*n* = 103) or negative (*n* = 117). In the ERG positive group, single ERG alteration was strongly associated with GS = 6 prostate tumors. The vast majority of low GS tumors had only a positive ERG immunostaining, probably due to *ERG* rearrangement. The double ERG positive/PTEN loss and the “triple hit” combinations were strongly associated with GS = 7 and GS ≥ 8 tumors, respectively. In contrast, in the ERG negative group, SLC45A3 and PTEN loss were not associated with any of the GS subgroups.

In GS ≥ 8 prostate tumors, we compared cases with two different Gleason pattern combinations: those in which the highest GS core did not contain any pattern 3 and those that did contain a pattern 3 component. Total ERG positive IHC (but not ERG as a single alteration) and PTEN loss were statistically associated with cases containing pattern 3. The “triple hit” phenotype (ERG positive/SLC45A3 loss/PTEN loss) was statistically associated with tumor foci containing pattern 3 areas as well. Thus, GS ≥ 8 PrCa with pattern 3 is associated with a subset of ERG positive cases, and GS ≥ 8 PrCa without pattern 3 is conversely associated with a subset of ERG negative cases. These findings seem to support the existence of two discrete pathways of prostate carcinogenesis: ERG positive cancer that progresses from pattern 3 towards pattern 5, and ERG negative cancer that more often arises as a “*de novo*” high grade tumor. PTEN and SLC45A3 alterations would be highly prevalent additional molecular changes associated with progression in ERG positive, but not in ERG negative cases.

In keeping with previous studies [12–15], our findings support the concept of *ERG* rearrangement being an early event in prostate carcinogenesis. Thus, the results of the present study seem to indicate that, in typical ERG positive low grade cases, *ERG* rearrangement would be the single main molecular event. PTEN loss would mark the transition to GS = 7 (GG2/GG3), i.e. the appearance of pattern 4, and loss of SLC45A3 expression would further determine progression towards highest grades (GS ≥ 8; GG4/GG5). Previous studies have described *PTEN* loss as a subclonal event after *ERG* gene fusion within a given established prostatic carcinoma clone [29]. In addition, *PTEN* loss in GS 6 biopsies has been found to identify a subset of prostate tumors with increased probabilty of upgrading at radical prostatectomy [31]. In a similar way, *PTEN* loss and chromosome 8 alterations in Gleason pattern 3 PrCa cores have been shown to predict the presence of un-sampled pattern 4 component [32]. Several papers also suggested that Gleason pattern 3 tumors containing pattern 4 differ at the genomic level from those having only pattern 3, that is true GS = 6 tumor [30–32, 43]. The results of a recent paper from our group [11], pointed towards the possible application of the “triple hit” IHC phenotype as an exclusion criterion in needle biopsy cases containing GS = 6 foci only. Finding the “triple hit” in such cases would probably mean that these foci are Gleason pattern 3 areas in a case already having unsampled pattern 4. The present results confirm these previous findings, indicating that the assessment of these three molecular changes could impact prognosis and therapeutic decision making in PrCa.

Perner *et al.* [33] hypothesized that SLC45A3 protein is a marker of prostatic differentiation, and hence SLC45A3 protein loss would be an independent sign of dedifferentiation of PrCa. According to our results, loss of SLC45A3 expression as part of the triple hit is associated with GS ≥ 8 containing pattern 3 areas, but when it is the only altered gene (with normal ERG and PTEN) it is independently associated with the opposite situation, i.e. GS ≥ 8 foci devoid of pattern 3 component. This could indicate that high GS prostate tumors without *ERG* rearrangement could evolve from an *ERG* independent pathway.

Finally, we have investigated the potential impact of our findings on PSA progression-free survival. Several previous papers have addressed different aspects of this relationship. Yoshimoto *et al.* reported that, taken together, *TMPRSS2-ERG* fusion and *PTEN* loss predict earlier biochemical recurrence [25]. Nagle *et al.* [44] found that *ERG* overexpression and *PTEN* deletion are associated with greater risk of the so-called capsular penetration. Moreover, Leinonen *et a.l* [45] reported that loss of PTEN expression was associated with shorter progression-free survival in ERG-positive, but not in ERG negative cases. Krohn *et al.* [22] found an association between *PTEN* genomic deletion and tumor progression and early PSA recurrence, in both *ERG* fusion-positive and fusion-negative PrCa. Very recent papers have shown that *PTEN* loss is independently associated with an increased risk of lethal progression, particularly in the *ERG* fusion-negative subgroup [46], and that *PTEN* deletion is significantly associated with extraprostatic extension, seminal vesicle involvement, and higher Gleason score. In addition, *PTEN* homozygous deletion was associated with worse post-operative recurrence-free survival [23]. It has also been shown that patients with ERG overexpression and normal PTEN had the best, and patients with ERG negativity and PTEN loss the worst biochemical recurrence-free survival [47]. In the present study, the number of aberrant events and the “triple hit” phenotype were strongly associated with a shorter time to PSA progression. ERG positive immunostaining (due mostly to a *TMPRSS2-ERG* rearrangement) and PTEN loss showed a trend but were not statistically associated with a shorter PSA progression-free interval. In the GS ≥ 8 PrCa, the “triple hit” was also statistically associated with PSA progression. In the GS = 6 tumors, the presence of isolated ERG alteration was associated with progression. In contrast with the results of Perner *et al.* [33], loss of SLC45A3 expression was not associated with progression in our series.

In conclusion, the results of the present study suggest that complementing the immunohistochemical assesment of ERG with SLC45A3 and PTEN expression status (“triple hit” phenotype) identifies PrCa subgroups with different molecular pathways, and, because of the relationship of this combination of markers with tumor grade and biochemical progression, they can be used to improve patient stratification, treatment and follow-up.

## MATERIALS AND METHODS

### Patients and tumor samples

Two-hundred and twenty prostate tumors were selected retrospectively from the files of the Parc de Salut MAR Biobank (MARBiobanc, Barcelona, Spain). All of them were FFPE. According to the International Society of Urological Pathology (ISUP) revised criteria [34], the Gleason scores (GS) of the prostate tumor samples were: 3 + 3 = 6 (*n* = 39), 3 + 4 or 4 + 3 = 7 (*n* = 103), and 4 + 4 or 4 + 5 ≥ 8 (*n* = 78). One of the comparisons among the different groups was based in subdividing GS ≥ 8 in two categories: those including pattern 3 areas (GS ≥ 8(3), *n* = 35) and the cases in which pattern 3 was not found in the core (GS ≥ 8, *n* = 43). On the other hand, according to the new Grade Group (GG) proposal by the WHO-ISUP 2016, consisting of five categories (GG1: 3 + 3; GG2: 3 + 4; GG3: 4 + 3; GG4: 4 + 4; GG5: 4 + 5 or 5 + 4 or 5 + 5), samples were classified as: GG1 (*n* = 39), GG2 (*n* = 81), GG3 (*n* = 22), GG4 (*n* = 33) and GG5 (*n* = 45).

### TMA construction

Nine tissue microarrays (TMAs) were constructed using a manual tissue arrayer (Chemicon ATA-100). Tissue cores of 1 mm in diameter were obtained from the most representative tumor areas of FFPE tissue blocks and were arranged in recipient TMA blocks. The TMAs contained cores from 220 radical prostatectomy specimens. For each case, 1–2 cores of the different Gleason score tumor areas were selected from haematoxylin-eosin (H&E) stained sections of donor blocks. From the nine resulting TMAs, sections of 3 μm were transferred to glass slides. H&E staining of the TMA sections were performed for diagnostic and grading confirmation and assessed by two expert pathologists.

### Immunohistochemistry of ERG, SLC45A3 and PTEN

The immunohistochemical expression of the three proteins was assessed in 581 cores derived from the 220 cases. Association between markers was assessed in individual cores. In 38 cases, only one core was available for the analysis. Immunohistochemical analysis was carried out using the primary rabbit anti-ERG monoclonal antibody (clone EPR3864, Epitomics, Burlingame, CA, USA), anti-prostein (SLC45A3) monoclonal mouse antibody (clone 10E3, Dako), anti-PTEN monoclonal mouse antibody (clone 6H2.1, Dako) and the Dako Envision+ System-HRP (Dako, Glostrup, Denmark). In addition, immunohistochemical analysis of SLC45A3 was also perfomed in whole sections from an independent series of radical prostatectomy specimens (*n* = 37). For the ERG immunostaining analysis, only two patterns of nuclear expression were considered: negative (without any detectable staining) or positive (with detectable nuclear staining). We used endothelial cells as internal positive control in each slide. For SLC45A3 and PTEN immunostaining analysis, a semiquantitative scoring system was applied: total loss of expression = 0, partial loss of expression = 1 and intense, homogeneous expression = 2. Adjacent normal tissue was used as an internal reference point for intensity scoring. For the purposes of this study we have considered as normal negative ERG immunostaining and homogeneous intense expression of SLC45A3 and PTEN.

### Fluorescence *in situ* hybridization (FISH) analysis of *SLC45A3*

To assess the genetic status of *SLC45A3* gene and to correlate with the immunostaining pattern of the protein, FISH analysis was carried out in an independent series of FFPE tissues from 37 prostate tumors. Rearrangement status was evaluated using break-apart probes. The bacterial artificial chromosome (BAC) clones RP11-326J24 5′/RP11-1143H2 3′were used for *SLC45A3.* We used consecutive sections for hematoxylin-eosin (H&E) and FISH in each of the samples. FISH technique and analysis were performed as previously described [11].

### Statistical analysis

Categorical variables are presented as frequencies and percentages, and quantitative variables as median and range. Pearson Chi-Square test or Fisher’s Exact test were used to assess the relationship between two categorical variables. For the different statistical analysis, expression of each of the three proteins was assessed either alone, this is withouth taking into account the status of the other two, or in all the possible different combinations: single alteration, this is considering only cases that had abnormal expression of one of the three genes and normal expression of the other two, as well as double and triple alterations.

Sensitivity, specificity, positive (PPV) and negative (NPV) predictive value for *SLC45A3* rearrangement compared to SLC45A3 immunostaining was calculated. Also, the Cohen’s Kappa (κ) coefficient was assessed.

The relationship with PSA progression-free survival was analyzed using Kaplan-Meier (Log-Rank) test in 214 patients for ERG, SLC45A3 and PTEN (6 cases in this series were lost for follow-up). Patients were censored at the time of their last clinical follow-up appointment or when an increase in serum PSA > 0.4 ng/ml was detected. A *p*-value less than 0.05 was considered to be statistically significant. Statistical analysis was performed using the SPSS statistical package version 15.0 (SPSS Inc, Chicago, IL, USA).
